# Temperature gradient of vertical air column in gravitational field

**DOI:** 10.1038/s41598-022-10525-0

**Published:** 2022-04-26

**Authors:** Han Mo Jeong, Sangyoun Park

**Affiliations:** Department of Chemistry, University of Ulsan, Ulsan, 44610 Republic of Korea

**Keywords:** Energy science and technology, Physics

## Abstract

The negative temperature gradient under gravity was observed with a vertical air column inside a practically insulated aluminum cylinder filled with sawdust. The temperature drop rate measured between 90 and 10 cm height positions was as much as 2.22 Km^−1^ when the diameter of the air column was 60 cm. This drop rate is much larger than the mean lapse rate of the earth’s troposphere (0.0045–0.0065 Km^−1^) and the previously reported experimental value (0.07 Km^−1^) by Graeff for the air column in a relatively small system. We proposed a kinetic model based on classical mechanics to account for this temperature drop, which is significantly larger than the values previously reported. The negative temperature gradient of the air column inside the cylinder showed a tendency to decrease sensitively as the positive temperature gradient of the ambient air outside the cylinder increased, although it was practically insulated. In addition, the temperature drop rate increased as the air column's diameter increased. These results suggest that as the size of the system increases, the external influence that relaxes the negative temperature gradient of the air column is diluted, and thus the negative temperature gradient becomes more pronounced.

## Introduction

In an isolated system, any heterogeneity of intensive properties, such as temperature, pressure, or density, becomes homogeneous over time, resulting in increased entropy according to the Clausius inequality^1^. However, some scientists suggested that the second law of thermodynamics could not hold unconditionally if an isolated system is in a field of force such as gravity^2–7^. Late in the nineteenth century, Loschmidt suggested that a vertical column of gas or solid would show a negative temperature gradient, being cold at the top and warm at the bottom if the system is insulated and left in a gravity field. His simple rationale is that with decreasing height, the potential energy of molecules decreases, which leads to an increase in the molecules’ kinetic energy (i.e., an increase in temperature). However, Boltzmann and Maxwell disagreed with the idea^8^.

The debate had not attracted much attention for more than a century, and no papers had published experimental results to substantiate Loschmidt’s idea. At the end of the twentieth century, A. Trupp published a paper summarizing and recalling this argument^7^. After several years, R. W. Graeff reported experimental results in which the negative temperature gradient was found in an insulated vertical tube filled with gases^9^ or water^10^, while the outside environment had a reverse temperature gradient. The negative temperature gradient measured was 0.07 Km^−1^ for air and 0.04 Km^−1^ for water. In Chapter 6 of the Reference 2, Graeff’s experimental results were summarized and evaluated^2^. At the end of this chapter^2^, Trupps’s experiments^5^ on the generalized Loschmidt effect applied to evaporation, which attempted to extend the Loschmidt mechanism to gases in other force fields, were also discussed.

In 2009, C. Liao published a paper on the temperature change according to the height of air column and of iron rod in a gravitational field. Both had a negative temperature gradient of 0.02  Km^−1^^11^. Moreover, in this paper, Liao introduced, summarized, and criticized the papers from various viewpoints on the temperature distribution of the vertical column of an adiabatically enclosed gas in a gravitational field. Most of these papers stated that the temperature of the air column is homogeneous with no temperature gradient^12–15^. On the other hand, Tolman predicted that there would be a negative temperature gradient based on the theory of relativity, but its magnitude would be too small to confirm experimentally^16–18^. To the best of our author’s knowledge, since the publication of Liao’s paper, no additional papers that measured and reported the temperature gradient of an adiabatic air column under gravity have been published.

The insulated air column, having a negative temperature gradient, can be utilized as a non-depleting, regenerable energy source because the temperature gradient tends to be kept in a gravitational field. In 1868, Maxwell proved that a perpetual motion machine of the second kind, which converts the energy of heat contained in the air into mechanical energy without requiring a second colder reservoir for the absorption of the refuse heat, would become possible if a vertical column of air subject to gravity had a temperature gradient with height^5^. In addition, a thermoelectric generator that converts heat into electrical energy can also be designed with the air column having a temperature gradient^19^. The larger the temperature gradient, the more power or electric current can be produced in these applications. Therefore, a large temperature gradient is required to put the air column into practical use as a valuable energy source.

This paper reports a significantly larger temperature drop with height than those reported was realized for a practically insulated air column in a gravitational field. For practically insulated air columns, it is not easy to completely block the influence from outside that relaxes the negative temperature gradient in the air column. We thought that these external influences would be diluted by increasing the size of the system. Therefore, in our experiment, air columns with several tens of centimeter diameters were used, whereas Graeff and Liao performed the experiments with small air columns having a diameter of 10 cm or less^9,11^. The effect of column diameter and column height on the temperature drop was scrutinized to confirm our thought. In addition, the effect of the changes in ambient temperature on the negative temperature gradient of the air column was investigated. In our experiment, the air column was filled with sawdust to minimize the convection in the system.

Graeff and Liao theoretically explained their results on the negative temperature gradient of the air column in the gravitational field as follows^9,11^. If a gas molecule moves to a higher position in a gravitational field without any intermolecular collision, the potential energy of the gas molecule will increase due to gravity, which results in a decrease in the kinetic energy of the gas. Thus, a gradient of mean kinetic energy and consequent negative temperature gradient will exist to the height direction when the molecular system is under gravity. The temperature gradient calculated based on this assumption could explain Graeff’s and Liao’s findings; however, it could not account for the significantly large temperature gradient observed in our experiment. Therefore, this paper explained our experimental results using our kinetic model based on classical mechanics.

## Methods

**Figure 1:**
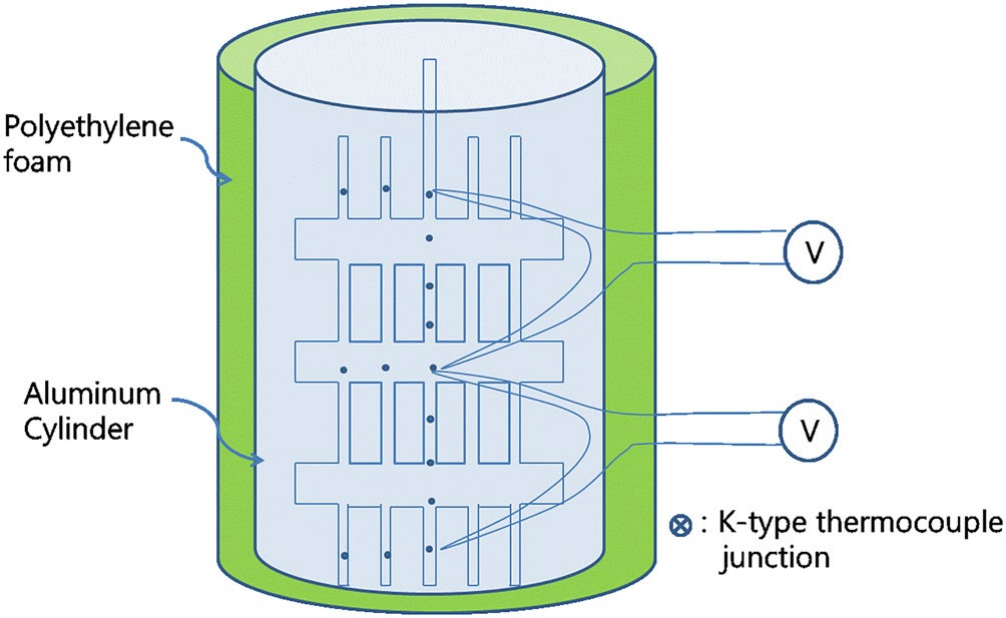
Schematic of the insulated air column to measure a temperature distribution.

This study measured the temperature gradient generated under gravity with a vertical air column in an aluminum cylinder (Fig. 1). The height of the aluminum cylinder was 120 cm, and the wall thickness was 0.4–3.0 mm. Three kinds of aluminum cylinders, having inner diameters of 26 cm, 45 cm, and 60 cm, respectively, were used to observe the effect of cylinder diameter on the temperature gradient. The aluminum cylinder was insulated by wrapping three times with a polyethylene foam sheet (PES) coated with a thin aluminum film. The thickness of PES was 0.5 cm. Both ends of the aluminum cylinder were sealed with polyethylene foam and additionally insulated with cotton wool. To minimize the relaxation of air temperature gradient in the column through the heat transfer by convection in the column, the cylinders were filled up to 100.0 cm height with sawdust of Douglas fir after drying for 30 days at room temperature. The sawdust, which has low thermal conductivity and does not rot easily because of its high carbon-to-nitrogen atom ratio, was sieved to have the sizes less than 2.5 mm. The apparent specific gravity of the sawdust was 0.24, and the water content of the sawdust measured from the weight loss after drying at 100 °C for 30 min was about 8.1%. The sawdust, pressed under its own weight during the 30 days measurement period, was lowered to the height of 99.5 cm from 100.0 cm.

The temperature difference between the lower and upper positions of the air column was evaluated from the potential difference measured at the two positions. The sensors were made by connecting 5 to 40 pieces of K-type thermocouples in series (0.127 mm in diameter, Omega Engineering Ltd.). The potential difference between the two positions was measured with Keithley 2700 Multimeter/Data Acquisition/Switch system and Keithley 2182A Nanovoltmeter (Keithley Instruments Inc., USA). The two junctions of each sensor were located at 10–30 cm, 10–40 cm, 10–50 cm, 50–60 cm, 50–70 cm, 50–80 cm, and 50–90 cm height positions from the bottom, respectively. The measurement was carried out for 30 days from June 18th, 2021, in Gyeongju, Korea. According to the datasheet of Keithley Instruments Inc., the error range of the instrument is 100 ppm or less of the measured value when the measured value is 10 mV or less. Furthermore, according to Omega Engineering Ltd., the error range of the K-type thermocouple is 0.4% or less of the measured temperature. Thus, most of the measurement error will be attributed to the thermocouple rather than Keithley’s instruments. Therefore, it is considered that the error is 0.002 K or less when the measured temperature difference is 0.5 K since the error of the thermocouple is 0.4% or less.

## Results and discussion

**Figure 2:**
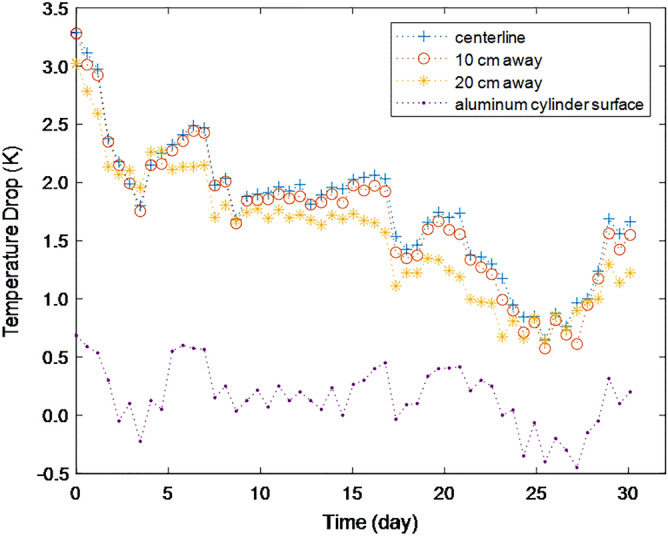
The temperature drops at 90 cm height compared to the temperature at 10 cm height for an air column of 60 cm diameter, measured with sensors located at three different radial positions for 30 days from June 18th, 2021, in Gyeongju, Korea.

Figure 2 shows the measured air temperature drop at 90 cm high position compared to the temperature of 10 cm high position in 60 cm diameter cylinder. In order to check the difference according to the sensor position in the radial direction, the sensors were installed at three radial positions: at the centerline, at 10 cm away from the centerline, and at 20 cm away from the centerline, and each was measured simultaneously. Figure 2 shows that the temperature drop is as much as more than 1.0 K during most of the measuring period, and the difference according to the sensor's location in the radial direction is not significant. As shown in Fig. 2, during most of the test period, the temperature of the top outer surface at 120 cm height of the aluminum cylinder was also lower than that of the bottom outer surface of the aluminum surface. This temperature drop change pattern of the aluminum cylinder surface over time was similar to the air temperature drops inside the cylinder, shown in Fig. 2. However, the magnitude of the temperature drop was much smaller. During the measuring period, the ambient air temperature varied in the range of 25–32 °C, and the temperature difference between the top and bottom positions of ambient air varied in the range 0.08–2.08 K (warm at the top, cold at the bottom). The fact that the temperature drop did not change significantly with the radial distance from the center suggests that there was hardly any fermentation of the packing sawdust that could affect the temperature distribution. If fermentation occurs, the core temperature is expected to be higher than that of the periphery. In another experiment, almost no change in appearance, temperature, odor, etc., was observed even when the sawdust mixed 1:1 with water was left for 1 month.

**Figure 3:**
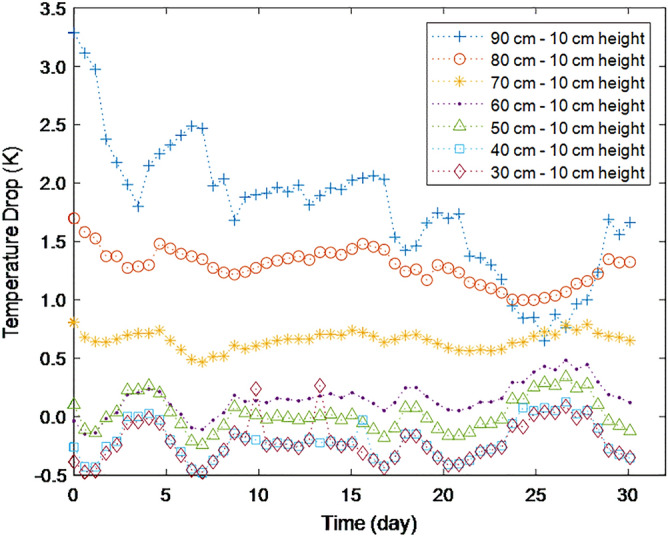
The temperature drops at various heights along the centerline compared to the 10 cm height for an insulated air column of 60 cm diameter, measured for 30 days from June 18th, 2021, in Gyeongju, Korea.

Figure 3 shows the temperature drop measured along the centerline at various heights compared to the temperature at 10 cm height. The average values of these temperature drops over the measurement period and the average values converted to temperature drop per meter of height (temperature drop rate) are summarized in Table 1. They show that the temperature drops, compared to that of the 10 cm height position, are observed at the height of 50 cm or more and the drop increases with the height. However, the temperatures increase at the heights of 30 cm and 40 cm. The temperature drop rates at high positions are much larger than the mean lapse rate of the earth’s troposphere measured (0.0045–0.0065 Km^−1^)^20,21^. Furthermore, these temperatures drop rates are much larger than the value, 0.07 Km^−1^, which Graeff experimentally measured for an air column of smaller size^9^.

**Table 1:** Temperature drops at various heights compared to the 10 cm height position and the drop rates for the insulated air column of 60 cm diameter under gravity.

Height (cm)	90	80	70	60	50	40	30
Average temperature drop (K)	1.78	1.29	0.65	0.15	0.02	− 0.21	− 0.21
Average temperature drop rate (K m^−1^)	2.22	1.84	1.09	0.30	0.04	− 0.69	− 1.07

In Eq. (1), M is the molar mass of air, g is the gravitational acceleration constant, C_p_ is the specific heat of air at constant pressure, and R is the gas constant. If it is assumed that the temperature drop of air with height (h) is due to the change of air’s internal energy (i.e., kinetic energy into potential energy), the temperature drop, ΔT at 90 cm from 10 cm, calculated with the Eq. (1) is 0.0078 K^21^. However, it can be seen that the measured ΔT value in Table 1 is 1.78 K, which is about 230 times the value estimated with Eq. (1).

1 $$\mathrm{M g \Delta h }=\mathrm{ C_{p} \Delta T }\approx \frac{7}{2}\mathrm{ R \Delta T}$$

In order to explain this significant temperature drop, we proposed a kinetic model of gas based on classical mechanics to estimate the energy flux due to gravity and the consequent temperature drop of a gas system in a gravitational field. In a closed cubic system of gas molecules under gravity (height ≈ ∞, number density of gas molecule ≈ 0), whose bottom wall is in thermal contact with a heat reservoir kept at a constant temperature and the other walls are insulated, the speed of a gas molecule will decrease as the molecule moves to a higher position from the bottom wall it collided with due to gravitational force and then finally will stop if there is no intermolecular collision. Thus, the z-component of velocity (v_z_) becomes v_z0_–gt at time t after a collision, where v_z0_ is the initial z-component of velocity. Therefore, the z-direction average kinetic energy of the gas molecule during the flight from the bottom wall to the stop position becomes $\frac{1}{6}$ mv_z0_^2^ as shown in Eq. (2) if there is no intermolecular collision, where m is the mass of a molecule and *T* = 2v_z0_/g. This suggests that the kinetic energy addition by gravity per flight after collision with the bottom wall is $\frac{1}{6}$ mv_z0_^2^ $-\frac{1}{2}$ mv_z0_^2^ =− $\frac{1}{3}$mv_z0_^2^. If one assumes that the gas molecules obey the Maxwell distribution of molecular speed after collision with the wall, the average kinetic energy addition by gravity after every collision will be − $\frac{2}{3}$kT (Eq. (3)), where k, T, and f(v_z0_) are the Boltzman constant, temperature, and the Maxwell one-dimensional distribution function of speed immediately after collision with the wall, respectively. Here, the f(v_z0_) is (m/2πkT)^1/2^ exp(-mv_z0_^2^/2kT) and v_z0_f(v_z0_) is the flux corrected distribution function of speed.

2 $${\int }_{0}^{T} \frac{\frac{1}{2}\mathrm{ m}{{\mathrm{v}}_{z}}^{2}\mathrm{ dt }}{T}={\int }_{0}^{T} \frac{\frac{1}{2}{\mathrm{ m}({\mathrm{v}}_{z0}-\mathrm{gt})}^{2}\mathrm{dt}}{T}=\frac{1}{6}\mathrm{ m}{{\mathrm{v}}_{z0}}^{2}$$

3 $$\frac {{{\int }_{0}^{\infty }-\frac{1}{3}\mathrm{mv_{z0}^2}}\mathrm{ v_{z0}{\mathrm{f}(\mathrm{v_{z0}})\mathrm{dv_{z0}}}}}{{\int }_{0}^{\infty }\mathrm{v_{z0}}\mathrm{f}(\mathrm{v_{z0}})\mathrm{dv_{z0}}}=-\frac{2}{3}\mathrm{KT}$$

The collision flux, the number of collisions divided by the wall area and the observation time, is $\frac{1}{4}$ cρ, where the average speed c is (8kT/πm)^1/2^ and ρ is molar concentration^22^. Therefore, the collision flux multiplied by the imaginary mean kinetic energy added by gravity during the flight, which we named as imaginary kinetic energy flux, can be expressed by Eq. (4), where ρ* is molecule number density.

4 $${\frac{1}{4}\left({8\mathrm{kT}}/{\mathrm{\pi m}}\right)^{1/2}}{\uprho }^{*}\left(-\frac{2}{3}\mathrm{kT}\right)=\frac{1}{4}(8\mathrm{RT}/\mathrm{\pi M})^{1/2}\uprho (-\frac{2}{3}\mathrm{RT})$$

Assuming that the molar concentration follows the Boltzmann distribution at all height ranges and the system's temperature is constant, the molar concentration at height h becomes ρ_0_ exp(− Mgh/RT), where ρ_0_ is the molar concentration at bottom. If one expands it as a Taylor series and takes only the first three terms for a small value of h, it becomes ρ_0_ (1—Mgh/RT + (Mgh/RT)^2^/2). Therefore, the difference of the net imaginary kinetic energy fluxes between the two vertically adjacent unit cubes, having 1 m edge length, under gravity becomes approximately $\frac{1}{4}$ c (− $\frac{2}{3}$ RT) ρ_0_ (Mg/RT)^2^.

If the net energy flux difference, flowing downwards and thus causing a negative temperature gradient, is balanced with the upward heat flow, and if the heat transfer by convection is negligible and the heat transfer by black-body radiation is negligibly slight because of low temperature, the following Eq. (5) holds for the temperature decrease (∆T) at 1 m height compared to that of the bottom, where κ is the thermal conductivity of air/sawdust mixture.

5 $$\frac{1}{4}\mathrm{c}(- \frac{2}{3}\mathrm{RT})\uprho_{0}(\mathrm{Mg}/\mathrm{RT})^2 \approx\upkappa \Delta \mathrm{T}$$

If the bottom temperature is 298.15 K and the pressure at the bottom is 1 atm, the ∆T at 1 m height is calculated with Eq. (5) as 3.98 K if the κ value of air, 0.026 Jm^−1^K^−1^s^−1^^23^, is used. It is calculated as 1.75 K when the κ value of sawdust, 0.059 Jm^−1^K^−1^s^−123^ is used instead. It can be seen that the temperature drop per meter observed in our experiment, 2.22  Km^−1^ (Table 1), lies between these two calculated temperature drops at 1 m height.

**Figure 4:**
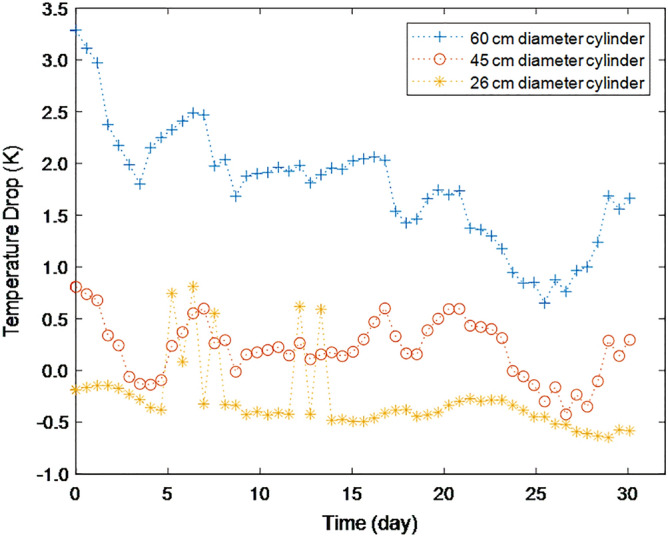
The effect of cylinder diameter on the temperature drops at 90 cm height along the centerline compared to the temperature at 10 cm height for an insulated air column, measured for 30 days from June 18th, 2021, in Gyeongju, Korea.

Figure 4 shows how the temperature drop at 90 cm height, compared to the temperature at 10 cm height, changes when the aluminum cylinder diameter is varied. Table 2 shows the average values for each cylinder. As the cylinder diameter decreases, the temperature drop is reduced, and it even has mostly negative values when the diameter is 26 cm. This result shows that system size (or the volume to surface area ratio) affects the temperature drop, increasing with increasing system diameter. This is thought to be because the external influence is diluted as the system size increases. For example, the effect of an aluminum wall to be a passageway for heat transfer and thus mitigate negative temperature gradients will be diluted as the system size increases.

**Table 2:** The effect of cylinder diameter on the temperature drop at 90 cm height compared to that at 10 cm height for an insulated air column.

Cylinder diameter (cm)	60	45	26
Temperature drop (K)	1.78	0.21	− 0.29

In order to investigate the effect of the ambient temperature on the temperature gradient in the cylinder, another experiment was performed. In this additional experiment, the aluminum cylinder having a height of 100 cm, an inner diameter of 22 cm, and a thickness of 1 cm was insulated with three layers of PES, an aluminum plate of 1.6 mm thick, and then an additional three layers of PES. The cylinder was filled with sawdust up to 100 cm. From March 2021 to July 2021, this study measured the ambient temperatures and the potential difference between 10 cm height and 90 cm height from the bottom inside the cylinder. Table 3 shows the average temperature drop of the air column at 90 cm height compared to the temperature at 10 cm height, calculated with the measured values for each month. In Table 3, it can be seen that as the surrounding temperature increases as the season changes from spring to summer, the increase in temperature with the height of the surrounding atmosphere becomes more pronounced. According to these changes, the degree of temperature decrease with height inside the cylinder gradually decreases, and temperature increases are observed instead from June. From these results, it can be seen that the temperature and temperature gradient around the cylinder affect the temperature gradient inside the cylinder, and the air temperature decrease with height inside the cylinder becomes more remarkable when the temperature increase of surrounding with height is minimized.

**Table 3:** The effect of the ambient air temperature on the temperature drop of the air column in a 22 cm diameter cylinder at 90 cm height position compared to the temperature at 10 cm height position.

Month	March	April	May	June	July
Ambient air temperature at 10 cm height (°C)	16.4	18.6	21.9	26.5	28.7
Ambient air temperature drop at 90 cm from that at 10 cm (K)	0.016	− 0.114	− 0.258	− 0.425	− 0.533
Air temperature drop in the cylinder (K)	0.023	0.017	0.002	− 0.016	− 0.031

## Conclusions

The experimental results of this paper inform the following facts.

1. The negative temperature gradient of the air column is generated under a gravitational field, and this temperature gradient changes sensitively according to the ambient temperature, even if it is practically insulated.
2. When the size of the air column is increased, the influence of ambient temperature that relaxes the negative temperature gradient inside the air column in the gravitational field can be diluted, so it is possible to enhance the formation of the negative temperature gradient inside the air column. Accordingly, in the experiment of this paper, an insulated air column with a diameter of 60 cm and a height of 90 cm exhibited a significantly large negative temperature gradient of 2.22  Km^−1^. This negative temperature gradient is much larger than the lapse rate of the earth’s troposphere measured (0.0045–0.0065  Km^−1^)^20,21^ and the experimental results by Graeff (0.07  Km^−1^)^9^ and C. Liao (0.02  Km^−1^)^11^ reported in the previous papers. This result suggests that a significantly large temperature difference can be obtained in a gravitational field with a large air column practically insulated, which can be utilized as a useful energy source.
3. Graeff and Liao theoretically described the results for the negative temperature gradient air column as follows: As air molecules move to higher positions, their potential energy increases and is compensated by a decrease in kinetic energy, resulting in a decrease in temperature and a negative temperature gradient. This rationale fits well with their experimental results, however, it could not account for significantly larger negative temperature gradient results. Therefore, we proposed a new kinetic model based on classical mechanics, which fits well with our experimental result.
